# Public Health in the Information Age: Recognizing the Infosphere as a Social Determinant of Health

**DOI:** 10.2196/19311

**Published:** 2020-08-03

**Authors:** Jessica Morley, Josh Cowls, Mariarosaria Taddeo, Luciano Floridi

**Affiliations:** 1 Oxford Internet Institute University of Oxford Oxford United Kingdom; 2 Alan Turing Institute London United Kingdom

**Keywords:** COVID-19, public health, misinformation, disinformation, infodemic, infodemiology, infosphere, social determinants of health, information ethics

## Abstract

Since 2016, social media companies and news providers have come under pressure to tackle the spread of political mis- and disinformation (MDI) online. However, despite evidence that online health MDI (on the web, on social media, and within mobile apps) also has negative real-world effects, there has been a lack of comparable action by either online service providers or state-sponsored public health bodies. We argue that this is problematic and seek to answer three questions: why has so little been done to control the flow of, and exposure to, health MDI online; how might more robust action be justified; and what specific, newly justified actions are needed to curb the flow of, and exposure to, online health MDI? In answering these questions, we show that four ethical concerns—related to paternalism, autonomy, freedom of speech, and pluralism—are partly responsible for the lack of intervention. We then suggest that these concerns can be overcome by relying on four arguments: (1) education is necessary but insufficient to curb the circulation of health MDI, (2) there is precedent for state control of internet content in other domains, (3) network dynamics adversely affect the spread of accurate health information, and (4) justice is best served by protecting those susceptible to inaccurate health information. These arguments provide a strong case for classifying the quality of the infosphere as a social determinant of health, thus making its protection a public health responsibility. In addition, they offer a strong justification for working to overcome the ethical concerns associated with state-led intervention in the infosphere to protect public health.

## Introduction

The internet’s capacity to generate and spread misinformation had already been identified and described 24 years ago [1]. However, it was the result of both the US presidential election and the UK’s referendum on European Union membership in 2016 that woke up civil society to the real-world effects of the online spread of false or inaccurate information (also known as *misinformation*) or deliberately misleading information (also known as *disinformation*; on the distinction between misinformation and disinformation [MDI] see [2] and more recently, in relation to health, [3]). As ever more political news and reporting has moved online—where network effects and a lack of gatekeepers mean that half-truths and mistruths can spread at greater speed and scale—the task of rapidly debunking false claims has been taken up by a growing army of fact-checking organizations. Additionally, social media companies have come under pressure to be more transparent about who has purchased specific political adverts and to provide consumers with “explanations” of the reasoning behind targeted adverts they see online [4]. These may only be small (and perhaps relatively ineffective) measures, but at least they show that there is growing consensus on the obligation of online service providers (OSPs) to take positive action to protect citizens and the democratic process [5]. Unfortunately, there have been almost no coordinated actions taken to tackle the equivalent issues associated with the online propagation of *misinformation* and *disinformation* as they relate to health. Throughout this paper, we use “health information” in a broad sense including medical information (eg, symptoms and treatments of specific diseases or injuries) and wellness information (eg, diet or fitness advice).

This lack of action is increasingly concerning, given the real impact of “the medical misinformation mess” [6]. “One day, being an inforg [informational organisms] will be so natural that any disruption in our normal flow of information will make us sick. Even literally.” That time has now come [7]. Yet, those actively seeking health advice and those browsing the web, social media, or even app stores for other reasons are faced with an almost constant barrage of medical news stories, social media posts, spurious website results, direct-to-consumer drug and medical adverts, and hospital and digital-health service marketing messages. Almost all of these are entirely inaccurate [6]. For instance, studies of vaccine-related internet content have shown consistently that most of this content is misleading and that false messages are more likely to be liked and shared than those that are accurate [8]. As a consequence of the lack of intervention in this state of affairs, hesitancy around getting vaccinated is now a major global health concern. At the same time, myriad online communities promoting self-harm, anorexia, and homeopathy now exist; unevidenced and unregulated apps are freely available for download; and the reckless promotion of fad diets and unproven wellness trends by celebrities on unregulated social media platforms is leading to the spread of various dangerous behaviors [9,10]. In short, as [11] states, “A child who needlessly experiences disabilities caused by measles, an adult who dies after stopping a statin despite having high-risk coronary heart disease, and a patient with cancer who ceases chemotherapy in favour of a bogus alternative are all victims of misinformation that is being promulgated on social media and other internet platforms.”

Events concerning the coronavirus disease (COVID-19) pandemic have only exacerbated the problem, to the extent that on February 15, 2020, the World Health Organization (WHO) Director-General Tedros Adhanom Ghebreyesus stated that: “We’re not just fighting an epidemic; we’re fighting an infodemic” [12]. Note that the word “infodemic” is not new. It was introduced in 2003 to refer to the spread of MID about severe acute respiratory syndrome [13].

Just like traditional political institutions, public health bodies underestimated the capacity of the web and social media to exert serious and potentially dangerous influence over health-related behavior [14]. This raises the following crucial questions:

- Why has so little been done to date to control the flow of, and exposure to, health MID online?
- In what circumstances is more robust action justified?
- What specific newly justified actions are needed to curb the flow of, and exposure to, online health-related misinformation and disinformation (OHMDI)?

In the following pages, we shall answer these questions in turn. Specifically, the section Missed Opportunities considers the early days of health information on the web, looking at why proactive attempts to govern better online health information were unsuccessful. The section Protecting the Individual Over the Group highlights how the criticism of public health interventions as paternalistic and antithetical to principles of bioethics has prevented further such attempts. The section Justifying Intervention discusses why these arguments are flawed in the specific context of OHMDI. The section the Infosphere as Social Determinant of Health provides a framework within which public health bodies may be able to act to tackle the OHMDI problem. The section Prevention, Protection, and Promotion: an Action Ontology provides a mode-of-action ontology for public health bodies. The section Actions and Agents considers the specific actions within this ontology that different actors in the system could take to tackle the problem. The last section concludes the article.

## Missed Opportunities

The quality of health information on the internet first became a major cause of concern for health care professionals, information specialists, and consumers of health care in the mid-1990s [15], when the web came to be portrayed by the medical community as a dangerous space that lacked the gatekeeping functions necessary to protect naïve health consumers [16]. The initial response to this mounting concern over the quality of online health information was a push from the medical community for greater regulation. However, when the rapid proliferation of content made it apparent that regulation would be unable to keep up, the focus of the community shifted toward market-based levers. This resulted in a proliferation of largely unsuccessful kitemarking, filtering, and accreditation schemes [17]. Textbox 1 summarizes four important examples of initiatives developed during this period.

**Examples of schemes concerning regulation of online health information.**

**Schemes**

- *The eHealth Code of Ethics*, produced by the Internet Healthcare Coalition in response to issues raised by the Food and Drug Administration in 1996 [18]
- *OMNI*, a search engine programmed to look solely for high-quality health care information designed to return only “validated” results [18]
- *The European Commission’s code of good practice for health websites*, published in June 2002 and based on the principles of transparency and honesty; authority, privacy and confidentiality; currency, accountability, and accessibility [19]
- The creation of the *Health on the Net Foundation* in May 1996, following the 1995 international meeting *Use of the Internet and World-Wide Web for Telematics in Healthcare*, which was charged with enabling the appropriate and efficient use of reliable health information online

Such attention from academia and supranational policy makers prompted the WHO to act, submitting a proposal to the Internet Corporation for Assigned Names and Numbers (ICANN) for the creation of a sponsored top-level .health domain. This proposal suggested that the WHO would, through consultation, develop a set of quality and ethical criteria that would-be .health sites would have to meet; it would ensure compliance by random checks conducted on approved sites and have an annual reregistration process [20]. The intention was not to police or regulate all health information on the web but to offer a reliable go-to domain to support users who wanted to narrow their search field to include credible sources only [21].

This idea was broadly supported by ICANN’s chairman at the time, Vinton Cerf, who stated that “we feel it would [have been] a great benefit to consumers for guaranteeing the quality of health and medical information on the web” and encouraged the WHO to pursue the idea further [22]. However, this support was not strong enough in the face of opposition from various stakeholders. They successfully argued that the web could not be policed, users were already sophisticated enough to recognize quackery [21], and no one body should assume the right to veto many thousands of websites [22].

These arguments against the need for a sponsored .health domain have been so influential that the WHO has been discouraged from bidding for the domain name again [23] and almost all other quality measures have also failed to gain traction (excepting the HONcode certification scheme). ICANN opened a new large-scale program to create multiple general top-level domains (gTLDs). In June 2011, the domains .health, .care, .diet, .doctor, .healthcare, .help, .hospital, and .med all went to the highest private bidder [24]. Mackey and Nayer [24] describe this process and fallout in detail. Textbox 2 shows the current owners of each health-related domain. There was no requirement for the domain purchasers to meet any specific criteria. For example, it is currently possible to make unrestricted purchases of potentially dangerous domain names such as suicidetips.health.

Such a hands-off approach to the governance of health-related domains suggests that the global community has reached the conclusion that the right strategy for improving the quality of health information on the internet is not content moderation, monitoring, or certification of reliable sources; instead, the focus should be on educating content producers and consumers [25]. This is the argument provided by Eysenbach [26] in a *JMIR* editorial when discussing the difference between moderating *content* and *source* quality (original emphasis):

No single body (let alone the domain registrar) should determine what is “correct” health information. It cannot be the goal to “censor” content or the messages on .health websites. It will always remain up to the website owners to ensure “message credibility,” and will always remain the responsibility of users to learn how to distinguish quality sites.

This argument of scale being a barrier to intervention has only become stronger with the advent of other sources of unregulated online health information, such as social media and mobile apps. The following section considers the ethical arguments that have pushed internet governance, medical device regulators, and public health bodies in this direction.

**Operators of health domains as of January 2020, according to the Internet Assigned Numbers Authority.**

**.health**

DotHealth LLC

**.care**

Binky Moon LLC (subsidiary of Donuts Inc)

**.diet**

Binky Moon LLC (subsidiary of Donuts Inc)

**.doctor**

Binky Moon LLC (subsidiary of Donuts Inc)

**.healthcare**

Binky Moon LLC (subsidiary of Donuts Inc)

**.help**

Unriregistry Corp

**.hospital**

Binky Moon LLC (subsidiary of Donuts Inc)

**.med**

Medistry LLC

## Protecting the Individual Over the Group

To understand the reasons why suggestions that online health information (encompassing all sources such as the web, social media, and mobile apps) should be subject to more stringent controls have typically failed, it is important to recall that public health is focused on the population at large and is primarily *preventive*, while clinical medicine is focused on the individual and is primarily *reactive* [27]. Thus, public health bodies seek to understand the societal conditions under which people can lead healthier lives to minimize those threats to health that can be averted or lessened *only* through collective actions aimed at the community [28]. These actions include, for example, disease surveillance, epidemiological modeling, national screening and vaccination programs, water sanitization efforts, and quarantining [28]. In short, public health officials take the position that society as a whole bears responsibility for the prevention of ill health. This recognizes that sometimes individuals acting alone are powerless to make the necessary changes and that only by acting together through public institutions can they protect the health of the communities to which they belong. This argument provides the philosophical justification for state interventions that override individual freedoms for the sake of promoting public health [29]. The same argument motivated the oft-quoted conclusion of the 1905 Supreme Court ruling *Jacobson vs. Massachusetts*:

The liberty secured by the Constitution of the US to every person within its jurisdiction does not impart an absolute right in each person to be, at all times and in all circumstances, wholly freed from restraint. There are manifold restraints to which every person is necessarily subject for the common good.

Although public health interventions that are justified on this basis are intended to be a form of collective problem solving, they are often perceived as being overly authoritarian [30]. As a result, although science has advanced to improve the efficacy of public health programs, public acceptance of them has declined [31] as the philosophy underpinning public policy has shifted from consequentialism, contractarianism, and communitarianism toward liberalism and principlism. Principlism is an approach to ethical evaluations and decision making that relies on the application of moral principles, rather than high-level normative theories such as virtue ethics, deontologism, or consequentialism [32]. It is popular in professional contexts but liberalism has prevailed in public health contexts because these alternative philosophies, all of which are central to public health, have been criticized for undermining the rights of the individual, as they rely on the idea that the end justifies the means, and for embracing an inherently paternalistic approach. Indeed, as reported by Buchanan [29], for many, the central concern of public health ethics is when it is justifiable to override individual freedom for the sake of public health. This is even a critical concern of the landmark Lalonde [33] report, which first recognized the role of factors other than the quality of the health care system in managing public health. It states: “The ultimate philosophical issue...is whether and to what extent the government can get into the business of modifying human behavior, even if it does so to improve health” [33]. Thus, the question for several philosophers working in this area, such as Dworkin in [34-36], is not whether public health interventions are paternalistic, but when are paternalistic interventions justified [29,37]?

These questions have been complicated as principlism, as a basis for bioethics in the clinical domain, has expanded into the public health domain, with a growing emphasis being placed on the principle of “autonomy.” As a result, public health policy has been pushed away from the idea that some aspects of health are outside of individual control, with the focus instead on encouraging citizens to take individual (and sole) responsibility for their health [16]. For example, Public Health England’s *Change for Life* program is based entirely around individuals taking action to improve their own health by eating better and taking part in regular exercise, but it does not include initiatives to improve access to resources that would enable these behavioral changes.

In this context, it has become ethically and politically difficult to argue in favor of tougher online health information controls. A website, social media post, or mobile app can be written by one person and read, commented, shared, downloaded, and edited by thousands. Intervening by, for example, automatically removing or flagging MDI would be perceived as a paternalistic (or even censorious) restriction on individual autonomy, particularly when the current overarching health policy paradigm is heavily infused with the (misguided) belief that information automatically leads to individual empowerment [38]. Furthermore, regulation of online health information is likely to be accused of being in conflict with the right to freedom of speech [17] and so harmful to the development of a pluralistic society. This is because pluralism, tolerance, and broadmindedness must go together, according to the European Court of Human Rights, and there must be room for individuals to express controversial opinions, including those about health [39]. In addition, in 2012, Internet Freedom was declared a human right by the United Nations (UN) Human Rights Council, which called on states to ensure that the rights to freedom of expression and information, as presented in Article 19 of the Universal Declaration of Human Rights, would be upheld online as well as offline [5].

As a consequence, although platforms including Twitter and Instagram do block specific hashtags (for example, #proana, an abbreviation of “pro-anorexia,” is not searchable on Instagram), public health bodies have thus far managed to justify only noncoercive state-level interventions focused on educating citizens. For example, throughout 2019, Public Health England, National Health Service (NHS) England, and the Department of Health and Social Care ran the #ValueofVaccines campaign with the intention of maintaining parental confidence in vaccines and shifting conversations away from antivaccination [40]. More coercive forms of information control are perceived to be neither necessary nor proportionate.

This is undeniably a formidable set of arguments to tackle. However, in the context of online health information, there are a number of pertinent and convincing objections. These are set out in the following section.

## Justifying Intervention

### Overview

The shift toward liberalism and principlism as the philosophical grounding of public policy, including public health policy, has hindered those who have previously argued in favor of tougher regulation of online health information. Even the call by the 66th World Health Assembly in 2013 for all health-related gTLDs to be used to promote public health and for member states to work with ICANN’s Government Advisory Committee to ensure proper governance for .health [24] was unsuccessful in overcoming the arguments that such intervention would be unnecessarily coercive. It *is,* however, possible to make the case for intervening against this hostile backdrop by focusing on the following four arguments: (1) education is necessary but insufficient, (2) precedent, (3) network dynamics, and (4) justice.

### Education Is Necessary but Insufficient

Education has always been, and will always be, a vital and ongoing part of public health campaigns. It will undoubtedly play a key role in improving the extent to which individual citizens are *resilient* and *resistant* to health MDI. However, there is mounting evidence that education alone is likely to be an insufficient “solution.” Pluviano et al [41], for example, conducted an experiment where beliefs in the idea of vaccinations being linked to dangerous side effects and intentions to vaccinate a future child were measured before and after an educational intervention. They found that, at best, the educational interventions were ineffective and, at worst, had the unintended opposite effect of reinforcing inaccurate beliefs and reducing intentions to vaccinate. This may well be because the web and social media create fertile conditions for the spread of postmodernist beliefs that question the legitimacy of science and authority, and reject the idea of a single “truth” [42]. This would not only help explain why those that spread and accept MDI are unlikely to be persuaded by evidence, facts, and reasoning but also indicate that relying on education alone is becoming less effective over time due to the nature of the environment it is trying to control.

### Precedent

Most of the arguments concerning whether it is acceptable for states to engage in what some may perceive as censorship to protect the public from potentially harmful information are not unique to health information. It is relevant, therefore, that there is precedent for taking a stronger approach to content moderation in areas other than health. For example, Zittrain and Palfrey [43], Brown and Cowls [44], and Macdonald et al [45] discuss various states that have successfully defied an early wave of “cyberlibertarianism” to block content in the name of national security or moral protection. Internet filtering that targets the websites of insurgents, extremists, and terrorists generally garners wide public support, as does the filtering of content that is perceived to be antithetical to accepted societal norms, such as pornographic content or hate speech [43]. Thus, all leading social media companies stipulate in their terms of service that terrorist content is forbidden and have, since 2016, collectively maintained a shared industry database of hashes (unique digital fingerprints) that identifies content produced in support of terrorism so that it can be removed as quickly as possible or, ideally, prevented from being posted at all. These interventions are far from unproblematic, but they clearly indicate that, in specific cases, for particular purposes, and within appropriate constraints, it *is* reasonable to make an ethically sound case for restricting the rights of individuals to post and access any sort of information unrestrictedly [44].

### Network Dynamics

When considering the dynamics of information spreading and persuasion online, and especially on social media, it is questionable whether taking a relatively *laissez-faire* approach to online health information is actually an effective approach to protecting autonomy and pluralism. Let us begin by considering how information sources are selected online.

Trust in a source of health information is determined by a complex set of interacting factors [46], but a particularly influential factor is *perceived credibility* [47]. In the offline world, this is largely controlled by the gatekeeping function performed by clinicians. In an online world, however, this gatekeeping function is removed. This means that a far greater burden is placed on individual internet users to make their own judgements about credibility and to determine which sources they trust [48]. Several studies, including a particularly compelling one by Chen et al [49], have shown that individuals with lower eHealth literacy lack the skills necessary to determine the credibility of the source accurately, thus placing their trust in inappropriate sources of information, like social media posts. One potential reason for this is that social endorsement (ie, likes and shares) acts as a signal of perceived trustworthiness to those with lower eHealth literacy [48,50-52]. Thus, if social endorsement is having at least a minor impact on the extent to which individuals trust health information that they read online, it is questionable whether they are *genuinely* making an autonomous decision about which information to treat as credible and act upon.

Building on the previous phenomenon, the algorithms driving both search engine results and social media feeds prioritize posts or websites that lead to greater engagement. Human nature means that often these are posts and sites that are more consistent with already held beliefs, emotive or controversial [53]. OHMDI is considerably more likely to meet these “criteria” than scientific evidence-based medical information, meaning that OHMDI is far more likely to benefit from algorithmic amplification than content produced by reputable health sources. Agents who deliberately try to manipulate or confuse debates about health care are well aware of this phenomenon and exploit it to their advantage [54].

The combination of these examples of network dynamics amplifying OHMDI provide a robust rebuttal against the argument that pluralism is a universal good. Although providing all views with an equal platform might be justifiable or even desirable in some contexts, the benefits of this—for example, ensuring that all perspectives are heard or providing individuals with the opportunity to develop their own opinions—are less likely to apply in health care. Unlike in politics, the widespread practice of evidence-based medicine means that there is often a high degree of consensus on most common medical questions (although this can change with time, and may be less true for more emergent fields). In some cases, the removal of gatekeepers and the presumption that all “voices” have an equal right to be heard has the potential to do considerable damage in the health care context, sometimes more than in the political context. This makes prioritizing diversity of opinions less justifiable and desirable, and instead creates the foundation for arguing that some “opinions” are more important than others and should (ethically) have a greater (rather than equal) opportunity to be heard. All beliefs are born equal, but some grow to become knowledge, whereas others remain mere opinions.

### Justice

The threat posed by OHMDI is more closely reminiscent of the threat posed by infectious diseases than of the threat posed by individual “unhealthy” behaviors. OHMDI acts as a pathogen and spreads like a virus through the internet and social media, exposing all those who are susceptible, not just those who have autonomously decided to seek out “alternative” information [55]. We saw that this has recently led to speaking of an “*info*demic” in the context of the COVID-19 *epi*demic (emphasis added). Protecting those who are more susceptible to OHMDI (often those who have defining characteristics also associated with poorer health outcomes) is more about meeting the other aim of public health interventions—reducing health inequalities—than it is about paternalistically deciding what “is best” for society. In other words, it is a matter of justice.

As the Marmot Review *Fair Society, Healthy Lives* made clear, health inequalities do not arise by chance. They cannot be attributed simply to genetic makeup, “bad” unhealthy behavior, or difficulties in accessing medical care. Instead, social and economic differences in health status are caused by social and economic inequalities in society. Health inequalities that are preventable by reasonable means are unfair and putting them right is a matter of social justice [56]. From this perspective, group-level interventions can still be respectful and protective of individual autonomy in a Kantian-Rawlsian sense (ie, as an integration of freedom and responsibility). Autonomous agents can accept moral constraints (provided they are transparent [29]) out of respect for others’ freedom, autonomy and dignity, and hence fairness and equal opportunity for all members of society [37].

Taken together, these four arguments—(1) that education is necessary but insufficient, (2) that there is precedent for acceptable state-led control of the internet content in other domains, (3) that network dynamics adversely affect the spread of accurate health information, and (4) that justice is best served by protecting those susceptible to OHMDI—justify working to overcome the ethical concerns associated with state-led intervention in the infosphere in the name of public health. Infosphere can be interpreted both minimally, as the whole informational environment, and maximally, as a synonym with reality. Infosphere here refers to the whole information environment, the whole system of information objects including all agents and patients, messages, their attributes, and mutual relations [57] (see also [58]). Yet, this justification in itself may not be enough to warrant attention from public health bodies if they do not see the infosphere as falling within their sphere of influence.

## The Infosphere as Social Determinant of Health

As discussed in the previous section, the controversy surrounding the debate over whether it is ethically acceptable or even preferable to limit individual rights to protect the group is certainly one factor hindering state-led intervention on OHMDI. However, public health policy makers are not typically afraid of controversy. Often, public health interventions, for example the UK’s sugar tax, require public health bodies to confront powerful private corporations and frequently face public backlash. Both the moves to ban smoking in public spaces and to make wearing a seatbelt a legal requirement in the United Kingdom were socially controversial at the time, and yet, both are now widely accepted social norms [59]. It is possible, therefore, that this controversy is not the primary source of public health bodies’ hesitancy. Instead, it may be that public health bodies have not intervened because they do not see online information control as being within their remit. Therefore, before we move on, we must consider the boundaries of public health bodies’ remit.

To do this, we can turn to the 1978 Declaration of Alma-Ata, which is generally considered to be a major milestone in the field of public health. It described public health as “a social goal whose realisation requires the action of many social and economic sectors in addition to the health sector.” Over time, these social and economic circumstances that together influence health came to be known as the *social determinants of health* (SDOH). The SDOH are alternatively referred to as the wider determinants of health. This approach to public health became a global commitment that themed the World Health Conference on October 21, 2011, when the WHO adopted the Rio Political Declaration on SDOH [60]. As a result, contemporary public health policies and strategies are determined by analyzing variations in SDOH and how these lead to inequalities in health care and threats to public health [61]. The problem for public health bodies operating today is that, although the SDOH approach provides global public health bodies with a much broader and more flexible remit than they had previously, the social determinants themselves have not been updated for the information age. Instead, almost all SDOH theories today are still based on the Dahlgren and Whitehead [61] model of health determinants developed for the WHO in 1991.

**Figure 1:**
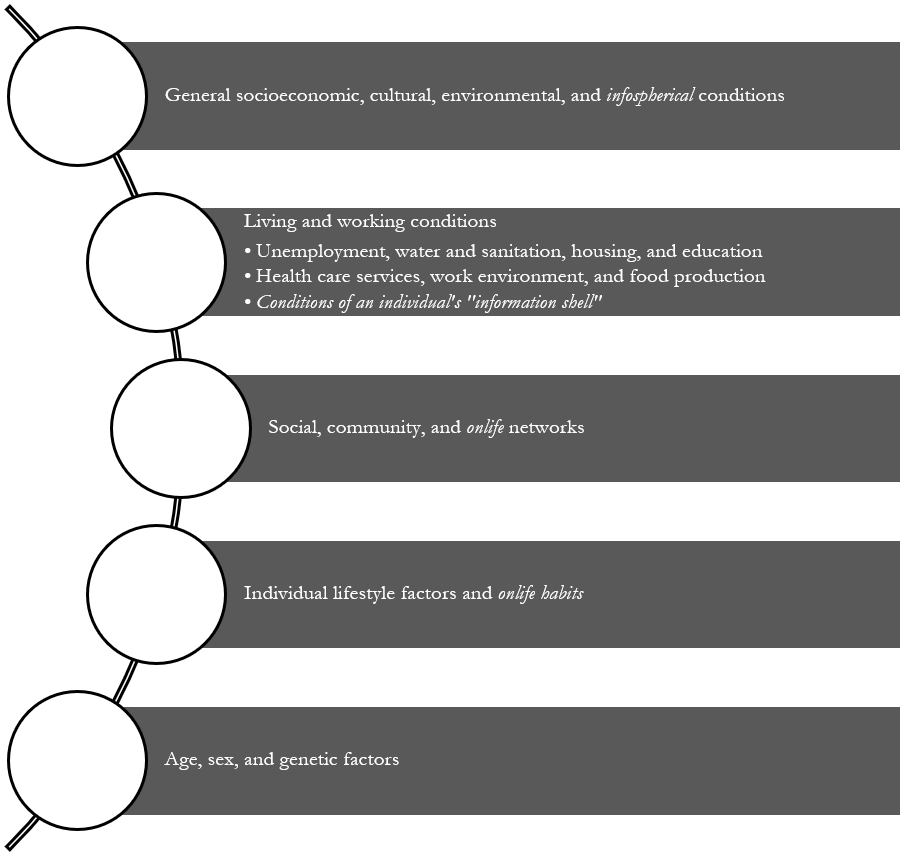
Onlife determinants of health adapted from Dahlgren and Whitehead (1991) [61] with italics highlighting new elements. The shell is a person’s personal world of information. It is constantly evolving through time and has a significant influence on a person’s behavior [57].

This model covers the *biosphere* and the *social sphere* but not the *infosphere.* This lack of attention to the infosphere is understandable, if one considers that this model was developed before the World Wide Web and declining costs of personal computers had enabled the global expansion of internet access [62], when social media and mobile apps did not exist, and when the dominant rhetoric was that online as a space had no bearing on physical realities. However, today the boundaries between online and offline are considerably less distinct and people living in the information age do live *onlife* [37,63]. It seems time that public health bodies accepted that the infosphere (encompassing all sources of online information) has a definite determining influence on the public’s physical health. Here we use the word physical to mean observable or demonstrable rather than “bodily” health. Undoubtedly, OHMDI will negatively affect individual’s mental health as well as their bodily health and both are equally important. Acknowledging this influence would make it possible to consider the infosphere as an SDOH and adapt the Dahlgren and Whitehead [61] model accordingly, as shown in Figure 1 adapted from [57,61].

In this model, the determinants at the top (general socioeconomic, cultural, environmental, and now *informational* conditions) are those over which public health bodies have the greatest degree of influence. In contrast, the determinants at the bottom (age, sex, and genetic factors) are those over which public health bodies have little to no influence. Thus, the model describes the remit of public health bodies and anticipates the range of activities these bodies might decide are necessary to improve the public’s health.

We can now conclude that not only is it possible to overcome the ethical concerns regarding individual autonomy vs group-level protection to justify government-led control of online health information, but also doing so definitely *is* within the remit of public health bodies. Having reached this conclusion, we must now move to consider *what* public health bodies can actually do to promote online health information.

## Prevention, Protection, and Promotion: an Action Ontology

In the previous sections, we had to distinguish between public health and clinical health before we could assess the ethical arguments used against state-led intervention in the infosphere and then identify the remit of public health before we could determine whether the conditions of the infosphere fell within it. Similarly, we now must consider *what* type of actions public health bodies can and do typically take before we can consider *how* these types of actions might be adapted for the infosphere context and the onlife experience.

Typically, public health bodies that operate at both a national or international scale conduct monitoring activities that enable them to identify public health *threats* such as air quality or pathogens that have the potential to cause harm. Depending on the threat level, responses can include issuing advice on *how* the public can keep themselves well or putting in place emergency measures such as the closing of airports to stop the spread of infectious disease in keeping with the 2005 International Health Regulations [64]. In short, almost all public health activities fall into one of the following three categories [65]:

1. Prevention: reducing the incidence of ill health by supporting healthier lifestyles
2. Protection: surveillance and monitoring of infectious disease, emergency responses, and vaccinations
3. Promotion: health education and commissioning services to meet specific health needs, for example, occupational health programs that promote self-care

Public Health England’s 2019 prevention guidance, for example, aims to address SDOH by breaking them down into the protective and risk factors [66] listed in Textbox 3.

**Positive and negative influences on a person’s health across the life course [66]. These are illustrative examples only, there are many other factors that could be listed in both categories.**

**Protective factors**

- Having a healthy and balanced diet
- An environment that enables physical activity
- Good educational attainment
- Being in stable employment with a good income
- Living in good quality housing
- Having networks of support including family and friends

**Risk factors**

- Smoking
- Adverse childhood experiences
- Crime and violence
- Drug and alcohol misuse
- Poor educational attainment
- Poor mental health

To make these types of activities relevant to the current discussion, we must take the previous step of arguing that, if the quality of the infosphere is an SDOH, then, poor information quality (OHMDI) is also a public health threat within the infosphere [8,67], just as poor air or water quality are public health threats within the biosphere. Thus, we must frame it as the potential source of an infodemic or a digital pandemic [14,68].

In the early 1990s, when (as discussed) the potential for this threat was first foreseen, there was insufficient evidence to support those claiming that online health information of poor quality could cause genuine harm to people’s health. This made it difficult to classify OHMDI officially as a threat to public health and use this as a means of demanding a public response [69]. This is no longer the case. Since the mid-2000s, the evidence demonstrating that the content people access online can affect significantly their health behaviors [70] has been growing. Researchers have connected proanorexia content with the rise in eating disorder incidence [71], antivaccination messages with a loss of trust in public vaccination programs [72], celebrity endorsements with mass uptake of fad diets and increased reliance on homeopathy and naturopathy over clinical intervention [9], and participation in specific chatrooms with suicidal ideation [73]. Furthermore, research has also shown that the creation of this potentially harmful content is not always unintentional. In 2007, the Wikipedia community identified a pharmaceutical company that was editing articles on Wikipedia and deleting side effects of specific medications [74]. Broniatowski et al [54] found that the Russian Internet Research Agency was using the hashtag #VaccinateUS to promote political discord. Therefore, it is clear that there is now an evidenced need to treat OHMDI as a public health threat and demand a robust and coordinated response [75], particularly as repeated exposure to potentially harmful information increases the risk that it poses [76].

Having completed this preliminary step, we can now return to the “prevent, protect, and promote” activities of public health bodies and clarify that:

1. Actions that lead to the automatic blocking of content classed as posing the highest risk to public health are *preventative*.
2. Actions that lead to the monitoring of content on social media or the wider web and the subsequent removal of potentially harmful information are *protective*.
3. Actions that improve access to and visibility of high-quality information are *promotional*.

Having established this ontology for the mode-of-action, we can now move on to examining the specific types of actions within each of these categories that different agents within the infosphere-as-SDOH may take.

## Actions and Agents

As the ultimate guardian of the public’s health, state-led public health bodies should take on overarching responsibility for the infosphere, ensuring that it flourishes and *protects* and *promotes* public health by *preventing* the appearance of threats in the form of OHMDI. The internet and the OHMDI circulating on it should be seen as the primary locus of this responsibility. In this respect, it should be noted that of the three categories for internet control identified by Eriksson and Giacomello [77], two (access to the internet and functionality of the internet) are outside of public health’s scope. However, it *is* possible and within scope for public health bodies to intervene in the third category of internet control: activity on the internet.

To make this clearer, it is possible to use the level of abstraction method of analysis. A LoA can be imagined as an interface that enables one to observe some aspects of a system analyzed while making other aspects opaque or indeed invisible. For example, one may analyze a house at the LoA of a buyer, of an architect, of a city planner, of a plumber, and so on. LoAs are common in computer science, where systems are described at different LoAs (computational, hardware, user-centered, etc). LoAs can be combined in more complex sets and can be, but are not necessarily always, hierarchical [78].

Public health bodies can regulate activity on the internet, by taking responsibility for interventions at the LoA *for* the web (LoA_FOR_) while enabling (and regulating) OSPs to take responsibility for interventions at the LoA *in* the web [5]. Responsibilities also present themselves at the LoA *on* the web, but as these responsibilities primarily concern themselves with controlling access to the metadata about user activities online [5], which public health bodies already do, for example, in cases of digital epidemiologic surveillance, we do not discuss these here. At the LoA_FOR_, public health bodies should develop programs of work focused on the following four areas identified by Chou et al [79]:

1. Defining the prevalence and trends of health MDI and identifying content for removal (protective monitoring)
2. Understanding what health MDI is shared and how it spreads so that it is possible to intervene earlier (preventive action)
3. Evaluating the reach and influence of high-risk health MDI (protective monitoring)
4. Developing and testing promotional responses

Collectively, these programs of work would enable public health bodies to monitor the most prevalent content being shared online, identify weaknesses in any current strategies, and detect new sources and causes of MDI before they result in significant harm [80]. To ensure the effectiveness of successful responses developed in (4) and based on the knowledge generated in (1), (2), and (3), public health bodies should leverage existing legal frameworks such as customer protection acts and laws governing health advertising [8] to determine when content is permissible (eg, the conditions under which celebrities or “influencers” are permitted to endorse and promote health- and wellness-related products such as “Skinny Tea”) and impose fines and other sanctions when these conditions are not met or when an organization, state, or person is found to be deliberately misrepresenting the scientific consensus with the intention of causing harm [81].

When these existing legal frameworks are found lacking, public health bodies should consider new, primary, or secondary legislation to ensure the protection of the public’s health [82]. They should also consider subsidies and tax breaks for OSPs that reflect social responsibility for public health in their terms of service *and* enforce these terms [81].

Finally, as Mackey and Nayyar [24] argued, global public health stakeholders should come together to rectify the mistakes of the past and recognize (as ICANN’s Independent Objector did) that the right to health is a fundamental human right, and this should include access to accurate health information. This means that .health and other health-related gTLDs should be protected by ensuring that the appearance of OHMDI is prevented and high-quality information is promoted. Collective action by then WHO, health-related UN organizations, and national governments should be demanded to secure a safe space for the health-related internet that abides by ethical principles, practices, and rules that honor public health interests [24] and ensure that information located within this space is authentic, truthful, accurate, clear, impartial, and evidence-based as much as possible [83].

Public health bodies can take the responsibility for creating the frameworks within which they and partnered private companies can intervene in the infosphere in the name of public health. Importantly, this must be done in a way that mitigates potential ethical risks related to information and data, such as privacy infringement, as much as is possible by encouraging public health bodies to consider how their interventions will affect the rights of both users and the environment [5]. At the same time, public heath bodies should regularly check that they are compliant with the International Health Regulations (2005) [64], that is, responding to a pressing public or social need pursuing a legitimate aim, being proportionate to the legitimate aim, and being no more restrictive than is required to achieve the purpose sought by restricting the right. They should also ensure that their actions are underpinned by the foundational values of public health ethics: transparency, confidentiality, and community consent [82].

By acting in this way, public health bodies can minimize both the harms of poor infosphere conditions and the ethical risks associated with public health policy. However, this does not mean that OSPs are discharged of all responsibility. OSPs should take responsibility for what it is *in* the infosphere and regulate it. They should discharge this responsibility, as stressed by Perakslis and Califf [11], by identifying, detecting, responding to, and recovering from OHMDI, and protecting accurate information (the five core cybersecurity functions listed by The National Institute of Standards and Technology Cybersecurity Framework [11]) from damage, destruction, misuse, and corruption.

There is already evidence that some OSPs are taking tentative steps in this direction. In November 2016, Facebook banned misinformation from advertisements on the site, including those promoting antivaccination messages. Pinterest has banned all antivaccination content outright. YouTube has removed advertising revenue and monetization from antivaccine channels and videos [81]. Twitter (at least in the United Kingdom) signposts users to the NHS website first when they search for derivates of “vaccine” on the website or app. The current COVID-19 pandemic has also sparked some specific action: Apple is rejecting all coronavirus-related apps that are not from governments or official health organizations; Google Play is blocking all searches for coronavirus; the UK government has set up a Rapid Response Unit to directly respond to false coronavirus narratives by, for example, issuing a rebuttal on social media or asking platforms to remove harmful content [84,85]; and, still in the United Kingdom, the Culture Secretary, Oliver Dowden, asked platforms to be more aggressive in contrasting conspiracy theories linking the coronavirus pandemic to 5G networks. Although positive steps, these measures are sporadic, *ad hoc*, unsystematic, and often far too narrow to make a considerable impact, especially when the far more extensive measures taken to tackle copyright-related, pornographic, or terrorist content are considered. Health-related information is hardly ever less, and often considerably more, important. Only by taking proactive, coordinated measures will OSPs, public health bodies, and app-store providers be able to stay one-step ahead of the rapidly evolving conditions of the infosphere and play their role in protecting public health [8].

## Conclusion

The WHO and the United Nations International Children's Fund may have become aware of the effects of deliberate disinformation campaigns before these spread online, with there being a notable coordinated campaign deployed to discourage women from receiving the neonatal tetanus vaccine throughout the late 1980s [86]. However, in the more than 3 decades since, frustratingly little has been done by public health bodies to tackle the internet’s ever-growing role in the “medical misinformation mess” [6]. As we have shown, this is also because, in the past, there has not been a strong enough case for prioritizing societal interests over individual rights [69] in this context. In the face of the “rising tide of medical misinformation” [75] and the adverse effect it is having on global health, a different approach should be adopted.

The problems with online health information, its quality, impact, and control, that we have discussed here are complex and multifaceted [87]. However, we have argued that the nature of the infosphere, its governance (or lack thereof), structures, affordances, and content is certainly contributing to new public health harms. We need to change the strategy. Before the situation becomes completely untenable and unmanageable, a robust and coordinated response from public health bodies, private corporations, and individuals is reasonable, ethically justified, and pragmatically necessary. It is also technically feasible. Although OSPs do bear responsibility as to the content circulating in the infosphere, it is up to public health bodies to shape, foster, and implement the necessary policies and actions to curtail the spreading of OHMDI.

If this joint response can be coordinated effectively, and the infosphere is appropriately recognized as a social determinant of (public) health and, therefore, a public good [75,88], then the twin goals of protecting the public’s health and reducing health inequalities can be supported. Identifying and implementing the most appropriate and efficacious interventions that fall within this framework may not be easy, but we should not let the scale of the challenge become a deterrent. Decisive action is needed, and it is needed as soon as possible.

## Abbreviations

COVID-19: coronavirus disease
gTLD: general top-level domain
ICANN: Internet Corporation for Assigned Names and Numbers
LoA: level of abstraction
LoA_FOR_: level of abstraction for the web
MDI: mis- and disinformation
NHS: National Health Service
OHMDI: online health-related misinformation and disinformation
OSP: online service provider
SDOH: social determinants of health
TLD: top-level domain
UN: United Nations
WHO: World Health Organization
